# Visual Stimulation Activates ERK in Synaptic and Somatic Compartments of Rat Cortical Neurons with Parallel Kinetics

**DOI:** 10.1371/journal.pone.0000604

**Published:** 2007-07-11

**Authors:** Elena M. Boggio, Elena Putignano, Marco Sassoè-Pognetto, Tommaso Pizzorusso, Maurizio Giustetto

**Affiliations:** 1 Dipartimento di Anatomia, Farmacologia e Medicina Legale and Istituto Nazionale di Neuroscienze, Università di Torino, Turin, Italy; 2 Scuola Normale Superiore, Pisa and Institute of Neuroscience, CNR, Pisa, Italy; 3 Dipartimento di Psicologia, Università di Firenze, Florence, Italy; Vrije University Amsterdam, Netherlands

## Abstract

**Background:**

Extracellular signal-regulated kinase (ERK) signalling pathway plays a crucial role in regulating diverse neuronal processes, such as cell proliferation and differentiation, and long-term synaptic plasticity. However, a detailed understanding of the action of ERK in neurons is made difficult by the lack of knowledge about its subcellular localization in response to physiological stimuli. To address this issue, we have studied the effect of visual stimulation *in vivo* of dark-reared rats on the spatial-temporal dynamics of ERK activation in pyramidal neurons of the visual cortex.

**Methodology/Principal Findings:**

Using immunogold electron microscopy, we show that phosphorylated ERK (pERK) is present in dendritic spines, both at synaptic and non-synaptic plasma membrane domains. Moreover, pERK is also detected in presynaptic axonal boutons forming connections with dendritic spines. Visual stimulation after dark rearing during the critical period causes a rapid increase in the number of pERK-labelled synapses in cortical layers I–II/III. This visually-induced activation of ERK at synaptic sites occurs in pre- and post-synaptic compartments and its temporal profile is identical to that of ERK activation in neuronal cell bodies.

**Conclusions/Significance:**

Visual stimulation *in vivo* increases pERK expression at pre- and post-synaptic sites of axo-spinous junctions, suggesting that ERK plays an important role in the local modulation of synaptic function. The data presented here support a model in which pERK can have early and late actions both centrally in the cell nucleus and peripherally at synaptic contacts.

## Introduction

The ERK/MAPK pathway has emerged as a central player in the signalling mechanisms underlying synaptic plasticity. Studies on the developing visual system have shown that ERK is strongly regulated by visual experience and that its activation is necessary for synaptic plasticity and ocular dominance plasticity [1]–[3]. Behavioral, electrophysiological and biochemical studies have suggested a role for ERK in plasticity also in other brain structures including the hippocampus, amygdala, striatum and cerebellum [4]–[6]. Although there is a general consensus about the crucial role of ERK in brain plasticity, little is known about the cellular mechanisms mediating its action(s).

It is presently believed that activated ERK translocates to the nucleus, where it targets several different regulators of gene expression, such as the transcription factors CREB and ELK-1 [4], and histone H3 [7], [8]. However, the discovery of other ERK targets in neurons, such as Kv4.2 [9], a potassium channel, synapsin I [10], a synaptic vesicle protein, Mnk-1 [11], a mRNA translation factor, and cytoskeletal proteins [12], has suggested that ERK may also be effective in the neuronal periphery and possibly in pre- and post-synaptic compartments. Indeed, signalling evoked by the ERK activator Ras has been found to be elicited by input activity in postsynaptic dendritic spines [13], and ERK inhibition affects structural plasticity and the synthesis of new proteins in a cell culture model of synaptic plasticity [14], [15]. ERK is also effective in the presynaptic compartment, where it regulates neurotransmitter release from excitatory terminals [16].

This wealth of biochemical and pharmacological information about the role of ERK in synaptic plasticity is not paralleled by a precise knowledge of the spatial and temporal regulation of ERK activity by physiological stimuli. This information is necessary to elaborate realistic models of the role of ERK in neuronal plasticity. To address this issue, we analyzed the spatio-temporal distribution of activated ERK in cortical neurons after visual stimulation by means of confocal and electron microscopy. We found that light exposure causes the activation of ERK both in the cell body of pyramidal cortical neurons and at the level of excitatory synapses, where phosphorylated ERK was found both in pre- and post-synaptic compartments. The kinetics of ERK activation were the same at the cell soma and at synaptic junctions. These data support a model in which pERK can have early and late actions at both nuclear and synaptic levels.

## Materials and Methods

Animals were used in accordance with protocols approved by the Italian Minister for Scientific Research. Control animals were exposed to a 12 hr light/dark cycle, with the light phase beginning at 6:00 A.M. All animals were killed at the same time of the day, between 8:00 and 9:00 A.M. Manipulations during dark rearing (DR) were performed in complete darkness using infrared viewers [1]. A total of 15 Long Evans Hooded rats inside the critical period (between 23 and 27 days of age) were used in this study. For preembedding immunoelectron microscopy, the animals were anaesthetised with an intraperitoneal injection of chloral hydrate and transcardially perfused with ice cold 4% paraformaldehyde, 0.1% glutaraldehyde and 1mM sodium orthovanadate in 0.1 m phosphate buffer (PB; pH 7.4). The same fixative without glutaraldehyde was used for confocal immunofluorescence. After perfusion, the brains were dissected and immersed in the same fixatives overnight at 4°C, after which they were washed several times in 0.1M PB.

### Light microscopic immunohistochemistry

Coronal vibratome sections (40 µm) were collected in phosphate buffered saline (PBS, 0.01 M, pH 7.4) and then treated free-floating. After a blocking step in a PBS solution containing 3% bovine serum albumin, 0.05% Triton X-100 and 10% normal goat serum (NGS), they were incubated overnight with a monoclonal antibody against the diphosphorylated form of ERK1/2 (Sigma, St. Louis, MO) diluted 1∶500 in PBS with 3% NGS and 0.05 Triton X-100. This antibody was raised against an 11 amino acid peptide (HTGFLT(Pi)EY(Pi)VAT) corresponding to the phosphorylated form of the ERK-activation loop, and does not recognize the non-phosphorylated or monophosphorylated forms of ERK, as well as the diphosphorylated forms of ERK-related kinases [17]. Subsequently, the sections were washed in PBS (4×10 min) and incubated 1 hour with goat anti-mouse biotinylated secondary antibodies (1∶250; Vector Labs, Burlingame, CA) diluted in 3% NGS and 0.05% Triton X-100 in PBS. For immunofluorescence, the sections were then incubated with streptavidin-Cy3 (1∶1000, Sigma), while for immunoperoxidase they were treated with the avidin-biotin complex (1∶100; VECSTAIN ABC kit, Vector Labs). The peroxidase reaction product was visualized by incubation in a solution containing 3,3′-diaminobenzidine (0.05% DAB in Tris-HCI, pH 7.6) with 0.01% H_2_O_2_ for 4 min. The sections were mounted on gelatine-coated glass slides and observed either with a light microscope (Eclipse 800, Nikon, Japan) equipped with a CCD camera (Axiocam HRc, Zeiss, Germany) or imaged with a confocal microscope (Olympus FV-300, Olympus Optical, Melville, NY).

### Electron microscopic immunohistochemistry

Vibratome sections (90 µm) of the primary visual cortex were cryoprotected in 30% sucrose in 0.1 M PB and then freezed and thawed to optimize the penetration of immunoreagents. The anti-diphosphorylated ERK1/2 antibody was diluted in the same dilution media used for light microscopy, but Triton X-100 was omitted from all solutions. Free-floating sections were blocked in 10% NGS for 2 hr and incubated in the primary antibody for 3 d at 4°C. After several rinses in PBS (4×10 min), the sections processed for pre-embedding immunoperoxidase detection were incubated for 2 hr at room temperature in biotinylated secondary antibodies, rinsed again in PBS (4×10 min), and transferred to a solution containing a biotin-avidin complex (Vector Labs). Finally, the sections were reacted in 3,3′-diaminobenzidine (0.05% DAB in Tris-HCI, pH 7.6) with 0.01% H_2_O_2_ for 4 min. The DAB reaction product was silver intensified and gold toned as previously described [18]. For pre-embedding immunogold, the sections were incubated for 2 h in FluoNanogold secondary antibodies (1∶100; Alexa Fluor-488 FluoNanogold anti-mouse Fab; Nanoprobes Inc., Yaphank, NY), diluted in PBS with 3% NGS, after which they were washed in PBS and postfixed in 1% glutaraldehyde for 15 min. After several rinses in double-distilled water, labelled sections were incubated in GoldEnhance EM (Nanoprobes) for 7 min to increase the size of nanogold particles to about 20–30 nm. All sections were postfixed with 1% osmium tetroxide (in 0.1 M cacodylate buffer) for 1 hr, dehydrated in a graded series of acetone (30–100%) and infiltrated with Epon 812 (Serva, Germany). The sections were then flat embedded between two pieces of Aclar film (Electron Microscopy Science, Hatfield, PA) and the resin was allowed to polymerize for 48 hrs at 60°C. Ultrathin sections were cut parallel to the surface of the vibratome sections with an ultramicrotome (Ultracut, Leica, Germany) and collected on single slot grids filmed with a pioloform solution. All grids were counterstained with uranyl acetate and lead citrate before being observed in a JEM-1010 electron microscope (Jeol, Japan) equipped with a side-mounted CCD camera (Mega View III; Soft Imaging System GmbH, Germany).

### Controls

To test method specificity in the immunohistochemical procedures for both light and electron microscopy, the pERK antibody was omitted. Under these conditions, no immunoreactivity was observed. Moreover, intraperitoneal injection (100 ml/kg) of the specific inhibitor of the ERK kinase MEK1/2 (SL327, Tocris Bioscience, UK) before light exposure completely abolished pERK immunolabelling (Fig. S1A,B), thus confirming the ability of the antibody to specifically detect the phosphorylated form of ERK in tissue sections.

**Figure 1:**
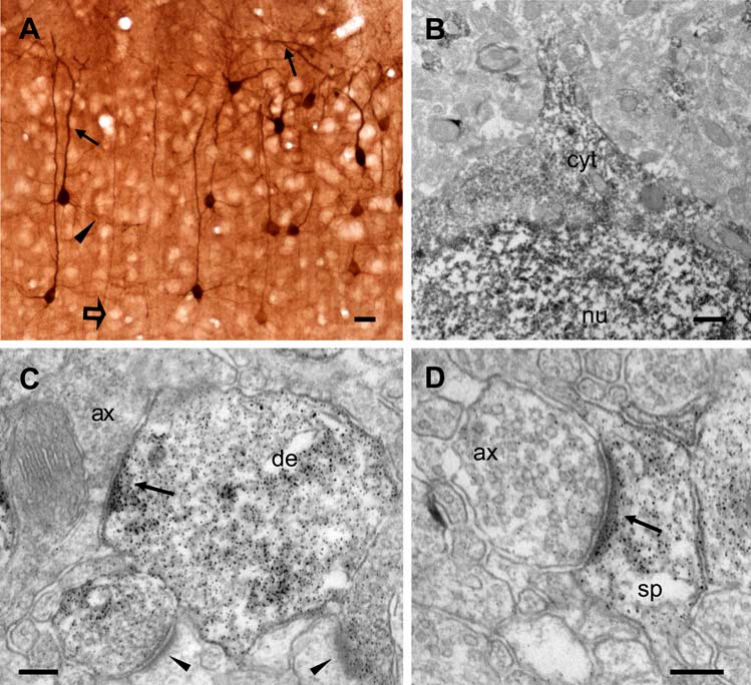
pERK immunoreactivity is present in a subset of axospinous synapses made by visual cortical neurons. (A) Immunoreactivity for pERK identifies individual pyramidal neurons in layers II/III of the primary visual cortex. Note that the large majority of cortical neurons are not labelled (open arrow). Both the apical dendrite (arrows) and basal dendrites (arrowhead) are clearly visible. (B–D) Electron micrographs revealing the subcellular distribution of pERK immunoreactivity in visual cortical neurons. At low power magnification, the immunoperoxidase reaction product is visibles in the cell nucleus (nu) and the cytoplasm (cyt) of a pyramidal neuron (B). C and D show pERK labelling at asymmetric synaptic contacts in cortical layer I. Arrows indicate an axo-dendritic (C) and an axo-spinous synapse (D) in which pERK immunoreactivity is postsynaptic. Note that pERK immunoperoxidase labelling is also present over the PSD of both contacts. In C, two presynaptically labelled axo-spinous contacts are also visible (arrowheads). Scale bars: A = 20 µm. B = 0.8 µm. C and D = 0.2 µm. de, dendrite; ax, axon terminal; sp, dendritic spine.

### Quantitative analysis

The number of pERK-positive axo-spinous synaptic contacts in layers I and II/III of the rat primary visual cortex was counted in three distinct animals for each of the four different visual stimulation conditions (DR, DR + 2.5 min of light exposure, DR + 15 min and DR + 40 min). In each experiment, comparisons were made with littermates that had been reared together and the final observer was always blind to the treatment. Synapses were counted in thin sections cut at comparable depths (100–500 nm) from the surface of the labelled vibratome sections, in order to minimize false-negative results caused by limited penetration of the antibodies. We systematically sampled all asymmetric contacts in non-overlapping electron micrographs taken in layer I (n = 20 digital images) and in layers II–III (n = 25), at a magnification of ×40.000. This led to an average of 190 asymmetric synapses analysed in each animal. The criteria for identifying asymmetric synapses were the presence of a presynaptic terminal containing synaptic vesicles, the parallel alignment of the pre- and post-synaptic membranes, and the presence of the postsynaptic density. A synapse was considered immunopositive when at least two gold particles were localized in either the pre- or the post-synaptic profile. The data were expressed as the percentage of pERK-positive profiles over the total number of synapses (mean±SEM of three rats). Statistical analysis was performed using a one-way ANOVA test with Origin 7 software (OriginLab Co., Northampton, MA). To determine the subsynaptic distribution of pERK in dendritic spine profiles, we divided the spine head profile into three distinct compartments: post-synaptic density (PSD), shell and core [19]. The number of gold particles and their association with each of these compartments were evaluated in 50 dendritic spines in DR and DR + 2.5 min animals. Labelling values were expressed as the density of gold particles found in each spine compartment.

## Results

### Immunogold detection of activated ERK at synaptic contacts in normally reared rats

Although several studies have analysed the expression of pERK in the brain, no information about its subcellular localization in neurons is currently available. We used immuno-electron microscopy to study the distribution of activated ERK in the primary visual cortex of juvenile (P27–P28) rats with a monoclonal antibody that is specific for the active, dually-phosphorylated form of ERK (ERK-1 and ERK-2, 44 kDa and 42 kDa, respectively). At the light-microscopic level, pERK immunoreactivity was evident in individual pyramidal neurons. A relevant number of labelled neurons were located in the more superficial cortical layers (II/III), whereas only a few pERK-positive neurons were found in the deeper layers. As shown in Figure 1, pERK immunoreactivity was not restricted to the cell body, but also extended to dendritic and axonal processes. Similar results (Fig. S1C) were obtained with a polyclonal antiserum raised against the dually-phosphorylated form of ERK (Cell Signalling, USA), thus confirming the specificity of our immunolabelling experiments.

**Figure 2:**
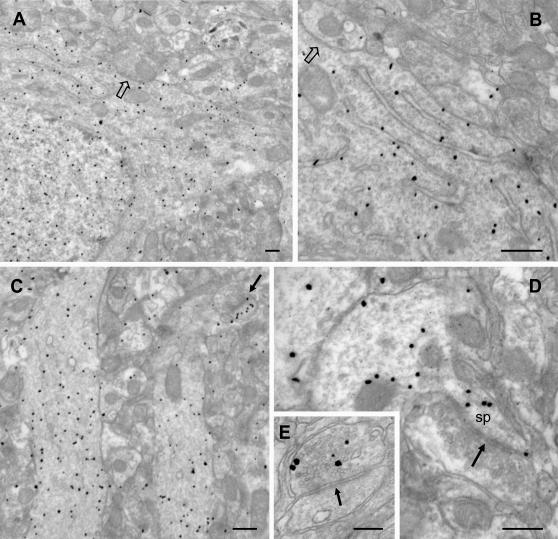
Electron microscopic immunogold localization of pERK in visual cortical neurons. (A) pERK immunogold labelling is visible in the cytoplasm and in the nuclear compartment of a pyramidal cortical neuron. (B) At higher magnification, gold particles decorate the external membranes of the rough ER. Note that symmetric synaptic contacts (empty arrows) appear to be mostly unlabelled. (C) Low power view of the neuropil of layers II/III showing intense pERK immunogold labelling in dendritic profiles. The immunoreactivity is distributed both in the dendritic cytoplasm and near or over the plasma membrane. An intensely labelled dendritic spine is also visible (arrow). (D) pERK immunogold signals in a dendritic process and a protruding spine (Sp) receiving an asymmetric synapse (arrow). Note that the presynaptic profile is completely unlabelled. (E) pERK labelling in a presynaptic terminal establishing an axospinous contact. Scale bars: A = 400 nm. B–E = 200 nm.

The ultrastructural analysis of immunoperoxidase labelling revealed strong pERK expression in the nucleus and in the perinuclear cytoplasm of layer II–III neurons (Fig 1B), as well as in dendritic processes (Fig 1C). Interestingly, pERK immunostaining was also found in asymmetric synaptic profiles, where it was localized in both pre- and post-synaptic terminals (Fig. 1C,D). Preembedding immunoperoxidase affords high sensitivity and efficient tissue penetration of the immunoreagents but suffers from diffusion of the peroxidase reaction product from the actual site of enzymatic reaction [20]. To improve the spatial resolution of pERK detection, we therefore turned to pre-embedding immunogold labelling (Fig 2A–E). The overall pattern of pERK localization obtained with this technique was similar to that observed with the immunoperoxidase method. However, the immunogold staining allowed us to resolve the presence of pERK in specific organelles and subcellular compartments. Gold particles were present at high concentration in the nucleus of pyramidal neurons and also decorated the external face of rough endoplasmic reticulum membranes (Fig. 2A,B). In the neuropil of layers I and II/III, pERK immunostaining was particularly enriched in dendritic processes, with gold particles distributed both in the cytoplasm and the submembranous compartment (Fig. 2C). Moreover, strong pERK labelling was observed in pre- and post-synaptic profiles (Fig. 2C–E), thus confirming the results obtained with preembedding immunoperoxidase. Labelling for pERK was localized both in the neck and the head of dendritic spines, as well as in proximity of the PSD (see below). In axon terminals, immunoparticles were closely associated with synaptic vescicles localized near the active zone (Fig. 2E). Although gold particles were occasionally observed in proximity of symmetric synapses on cell soma (Fig. 2A,B) or dendritic shafts, no enrichment of pERK labelling was specifically observed for this type of synaptic contact. Thus, our data are consistent with a role of ERK signalling at excitatory synapses as well as in the cell nucleus.

### Visual stimulation activates ERK at axo-spinous synaptic contacts

ERK can be activated in pyramidal cortical neurons by visual stimulation [1] and is involved in experience-dependent plasticity of the developing visual cortex [2]. To investigate whether visual experience can regulate ERK phosphorylation at synaptic junctions, we analyzed pERK immunogold staining in rats exposed to light for 2.5 minutes after a three-day period of dark rearing (DR). In order to avoid false negative synaptic labelling, in an initial phase of this study we sought to validate pERK immunogold labelling on consecutive thin sections. We found that the large majority of pERK-positive synaptic profiles were labelled in at least three consecutive sections (Fig. S2A,B), indicating that our method detected pERK with high efficiency at synaptic sites and that the amount of pERK-positive synapses can be reliably estimated with a single-section analysis.

**Figure 3:**
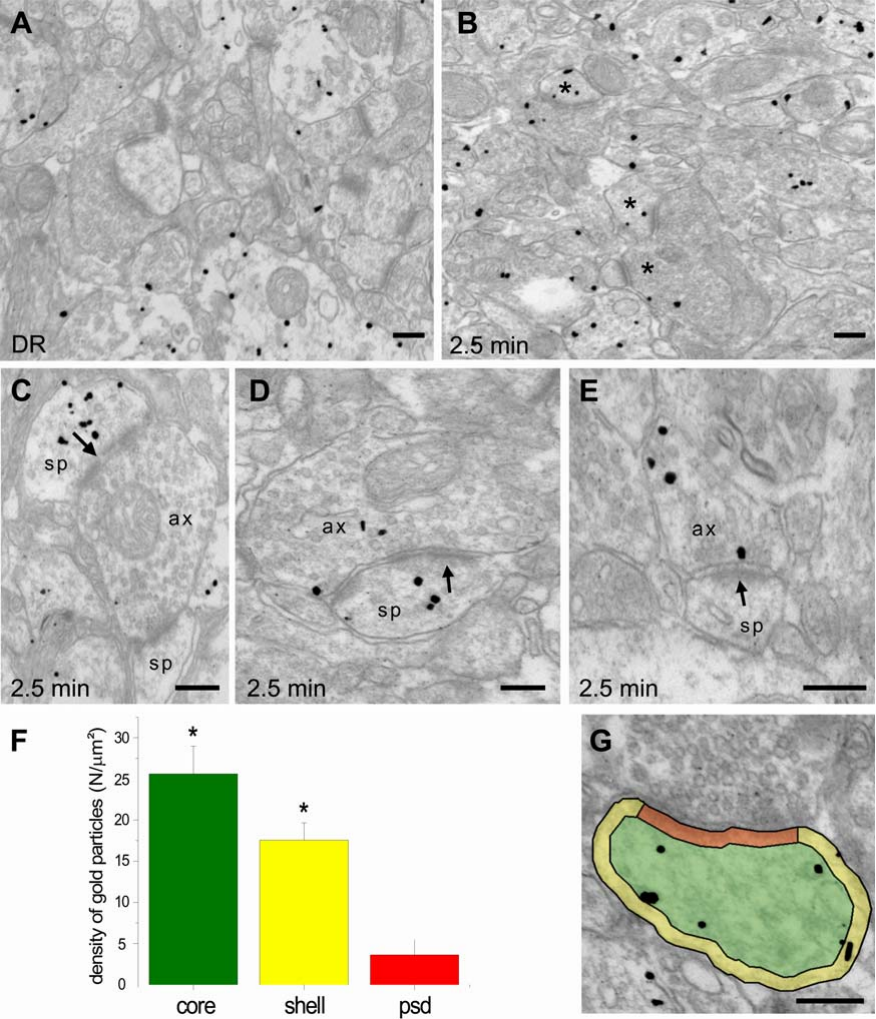
Light exposure increases the number of pERK-positive axospinous contacts but does not modify its distribution within dendritic spines. Rats were placed in complete darkness for three days at the beginning of the critical period for ocular dominance plasticity (P23–P27) and sacrificed in the dark (A) or after exposure to light for 2.5 minutes (B). Light exposure (B) caused a dramatic increase of the number of gold-labelled asymmetric contacts (asterisks) in layer I of the visual cortex. In some of the labelled synapses, gold particles were present exclusively either in postsynaptic spines (C) or in presynaptic terminals (E), whereas other synapses showed both pre- and post-synaptic labelling (D). (G) To analyze the subsynaptic distribution of pERK in the spine compartment, dendritic spines (n = 50) were divided into three sub-compartments: PSD (red), shell (yellow) and core (green). Histogram in F shows the density of gold particles (number of particles per µm^2^) found in each sub-compartment. One-way ANOVA; * = p<0.01. Ax, axon terminal; Sp, dendritic spine. Scale bars: 200 nm.

**Figure 4:**
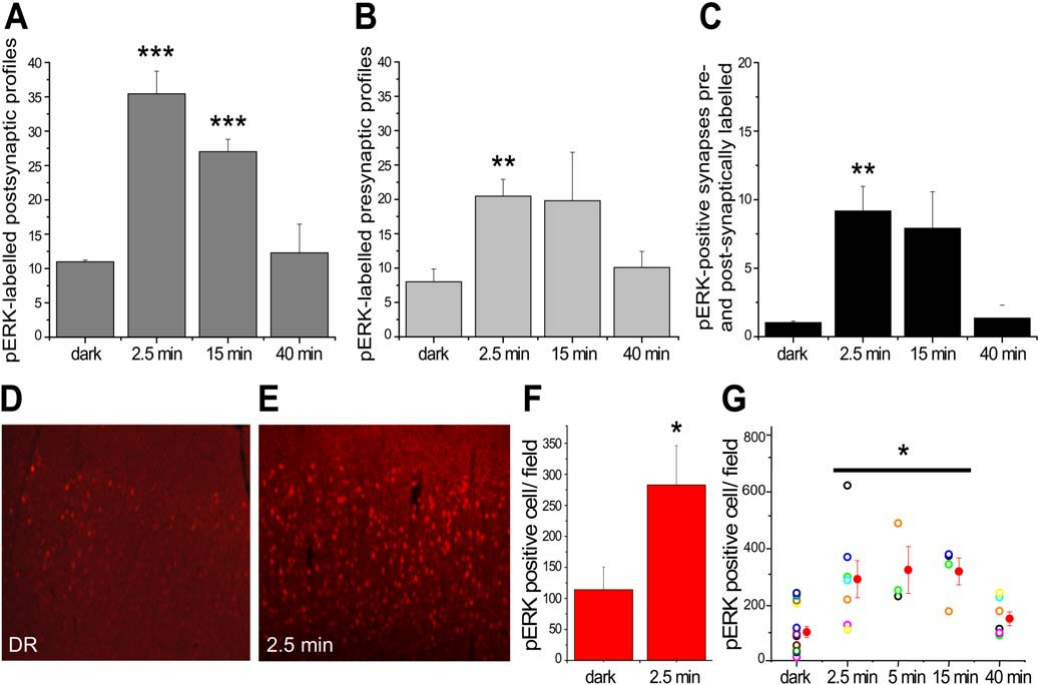
Temporal kinetics of ERK activation in synaptic and somatic compartments after visual stimulation. Histograms in A and B show the percentage of pERK-positive synaptic profiles (A, postsynaptic; B, presynaptic) out of the total number of synapses that were randomly sampled. (A) ERK activation in postsynaptic profiles was significantly elevated after 2.5 minutes of visual activity, remained stable for 15 minutes and returned to baseline/DR conditions after 40 minutes. (B) A similar temporal profile of ERK activation was observed in presynaptic profiles. Histogram in C show the percentage of pERK-positive synapses showing immunogold labelling simultaneously in the pre- and post-synaptic profile. (D,E) Confocal images of pERK immunofluorescence in layers II/III of the visual cortex of rats that were dark reared for three days and sacrificed in the dark (D) or after 2.5 min of light exposure (E). Note that light exposure causes a dramatic increase in the number of pERK-immunopositive neurons. (F) Quantitative analysis revealing that light exposure causes an almost three-fold increase in the number of pERK-positive neurons in the cortex of light exposed animals compared with DR rats. Data in G are partially re-plotted from [1]. The histograms show the time course of light-induced ERK activation in the cell soma and nuclei of visual cortical pyramidal neurons. One-way ANOVA; *** = p<0.01; ** = p<0.02 ; * = p<0.05; n = 3 rats for each experimental group.

Visual stimulation caused a dramatic increase in the number of axo-spinous contacts positive for pERK in layer I and layer II–III of light-exposed rats (fig 3B) with respect to DR animals (fig 3A). By examining consecutive sections, we found that in some synapses pERK staining was localized either pre- or post-synaptically, whereas in other synapses pERK immunoreactivity was present in both pre- and post-synaptic profiles (Fig. 3C–E). The number of pERK-positive presynaptic terminals was significantly more elevated in visually-stimulated rats (Fig. 4B) than in DR rats (8±1.9% in DR vs. 20.4±2.5% in DR + 2.5 min light; p<0.02). Similarly, pERK-positive dendritic spines were increased significantly (Fig. 4A) by visual stimulation (11.0±0.3% in DR vs. 35.4±3.3% in DR + 2.5 min light; p<0.01).

Cytoplasmic proteins, membrane proteins and protein components of the PSD are potential substrates of activated ERK in dendritic spines [4]. To better understand ERK function at postsynaptic sites in response to physiological stimulation, we analysed its distribution within dendritic spines of light exposed animals. Spine profiles (n = 50) were divided into three distinct compartments (PSD, shell and core, Fig. 3G) and the density of immunolabelling was determined in each compartment [19]. The results of this analysis revealed a preferential association of pERK with the shell and the core of the spine head, whereas pERK was less frequently localized in the PSD (Fig. 3F). Similar results were obtained in DR animals (data not shown). Therefore, visual experience increases the number of presynaptic terminals and dendritic spines containing the activated form of ERK without apparently affecting its ultrastructural localization within the dendritic spine.

### Rapid and transient activation of ERK in the neuronal soma and in synaptic contacts

It has been proposed that the early effects of ERK in synaptic plasticity are mediated by its action in the cell periphery, whereas the late effects involve the translocation of pERK into the nucleus [21]. Therefore, visual activation of ERK in cortical circuits may show different kinetics in different subcellular compartments. To assess the temporal dynamics of ERK activation at synaptic sites, we investigated the distribution of pERK immunogold labelling at different times after visual stimulation (2.5, 15, and 40 min). In addition, in a separate set of experiments we used laser scanning confocal microscopy to determine the kinetics of ERK activation in the cell body and nucleus of cortical neurons. Figure 4A shows that the percentage of pERK-positive dendritic spines remained significantly higher than control values between 2.5–15 min after visual stimulation and returned to DR levels after 40 min. At all time points, the percentage of axon terminals stained for pERK was lower than that of dendritic spines (Fig. 4B). However, the curve of ERK activation was similar in the two synaptic compartments, with an increase between 2.5 min and 15 min, and a return to basal levels after 40 min. Furthermore, we observed a marked increase of the proportion of synapses showing pERK labelling in both the pre- and post-synaptic compartment after visual stimulation (1.0±0,1% in DR vs. 9.2±1.8% in DR + 2.5 min light; p<0.02; Fig. 4C). Interestingly, the same temporal profile of ERK activation was observed in the cell soma (Fig. 4C–F): the number of pERK-positive neurons in layer II/III showed a ∼3-fold increase after 2.5 min of visual stimulation (101,5±18,9 cell/field in DR vs. 283±63,3 in DR + 2.5 min light; p<0.05), remained elevated at 15 min, and returned to the levels of DR animals after 40 min (Fig. 4F; see also [1]). Thus, our *in vivo* analysis shows that the synaptic and somatic pools of ERK are activated with similar kinetics after physiological stimulation.

## Discussion

In the present study we describe for the first time the ultrastructural localization of activated ERK in neurons. Using high-resolution immunolabelling, we show that visual stimulation evokes a robust activation of ERK at asymmetric synaptic contacts in layers I–II/III of the primary visual cortex. The activation of ERK at synapses is rapid and transient and occurs in both the pre- and post-synaptic compartment. This observation, together with previous findings showing that ERK inhibitors block visual cortical plasticity and cortical LTP [2], suggest that visual cortical plasticity likely depends on ERK activation at excitatory synapses. Moreover, our data show that the kinetic of ERK activation at synaptic sites parallels that in the neuronal cell body. This indicates that the differences between the early and late effects of ERK cannot be ascribed to a rapid and transient activation of ERK at synaptic sites followed by a slower and sustained induction in the cell soma, but that ERK activation in synaptic compartments can also be sustained.

### Local activation of ERK at excitatory synapses

Previous studies have shown that ERK plays a fundamental role in developmental plasticity of the visual cortex [22]. Activity-dependent potentiation of synaptic transmission in visual cortical slices is blocked by compounds that inhibit ERK activation [2]. In vivo, ERK inhibition prevents the effects of monocular deprivation on ocular dominance of visual cortical neurons [2], whereas its activation is strongly regulated by visual experience [1] and is necessary for transcriptional activation of several genes [23]. The subcellular localization of activated ERK and its precise role in synaptic plasticity have not been defined yet. The results of the present study suggest that the role of ERK in visual cortical plasticity is not limited to the regulation of gene expression, but also involves local mechanisms at the synaptic level. Light-activated ERK could contribute to the dynamic regulation of spine structure, a process that is thought to be at the basis of experience-dependent plasticity of the visual cortex [24]–[27]. The action of ERK could include the regulation of proteins involved in spinogenesis such as spinophilin [28], a newly discovered synaptic target of ERK signaling.

The contribution of ERK in synaptic plasticity extends to several other brain regions, including the hippocampus, amygdala and striatum [29]–[40], and it is believed that the long-term effects of ERK are due to its translocation into the cell nucleus and the regulation of gene transcription [21]. A point that needs to be addressed in future investigations is whether the synaptic activation of ERK that we have observed in the visual cortex can be generalized to other brain structures. According to a recent study, a form of hippocampal-dependent fear memory is associated with a long-lasting increase in pERK immunoreactivity in stratum radiatum of the hippocampal CA1 region [41]. These authors found that ERK was activated in dendritic profiles as well as in punctate structures that were closely associated with synaptophysin-positive boutons. Although additional studies are needed to fully understand the cell biology of ERK signalling in the CNS, it is possible that the local activation of ERK at synaptic sites represents a general mechanism in synaptic plasticity in different brain regions.

### Potential targets of local activation of ERK

What could be the role of the local activation of ERK at synaptic sites? In the cell periphery, ERK activity may target multiple substrates in the synaptic and dendritic compartment, and therefore contribute to the initial steps that lead to long-term synaptic changes [4]–[6]. Despite the function of ERK in synaptic plasticity has received considerable attention, whether and how ERK activation targets the pre- or the post-synaptic compartment is still an unresolved question. In this study we observed that brief light exposure triggers a rapid and strong activation of ERK in axon terminals and dendritic spines, suggesting that activity-dependent ERK phosphorylation may regulate the function of proteins located both in the pre- and post-synaptic compartments. Moreover, the number of synapses showing pERK labelling at both pre- and postsynaptic sides was increased after visual stimulation, although the incidence of concurrent pre- and post-synaptic labelling was not statistically different from what could be predicted assuming an independent regulation of ERK in the pre- and post-synaptic compartments. Nevertheless, given that synapses labelled on both sides represented ∼9% of total number of contacts, our data raise the interesting possibility that ERK could play a role in matching pre- and postsynaptic changes during long-term modifications of synaptic efficacy [42].

One interesting finding of our study is that pERK seems to be preferentially localized within the core and the shell of the spine, whereas it seems to be scarcely associated with the PSD. One of the potential postsynaptic targets of pERK is the voltage-dependent K^+^ channel (K_v_4.2), a regulator of dendritic propagation of action potentials [43], [44], whose phosphorylation increases at ERK sites during LTP [45]. Interestingly, an ultrastructural analysis revealed that in pyramidal neurons of the visual cortex K_v_4.2 is often localized along the non-synaptic membrane of dendritic spines as well as in postsynaptic membranes of GABAergic synapses [46]. Indeed, the immunogold localization of pERK in the shell of the spine is consistent with the idea that ERK could contribute to regulate K_v_4.2 channel function. ERK activation in dendritic spines could also be involved in regulating the insertion of AMPA receptors into the postsynaptic membrane. Recently, Malinow and colleagues (47) showed that ERK is directly involved in an increase of AMPA receptors-mediated synaptic transmission and in the synaptic delivery of AMPA receptors following LTP [47]. Our immunogold data showing that pERK is present in the core of dendritic spines, where AMPA receptors are accumulated in subsynaptic endosomes stores [48], support the role of ERK signalling cascade in the transport of AMPA receptors to the postsynaptic membrane.

In addition to the postsynaptic activation, we found that visual stimulation leads to a very rapid increase of the number of pERK-immunoreactive axon terminals, suggesting that visually-induced activation of ERK might regulate synaptic transmission and plasticity at the presynaptic level. While the precise role of pERK in axonal boutons is still unclear, it has been shown that ERK may act to phosphorylate synapsin I, a phosphoprotein that regulates the binding of synaptic vescicles to the actin cytoskeleton in an activity-dependent manner [49]. By phosphorylation of synapsin I at specific sites, ERK may regulate the proportion of vesicles in the nerve terminal that are available for rapid release [10] and modulate neurotrasmission [16].

Our study might also suggest that pERK could represent a mark of previously activated synapses, both at the pre- and at the post-synaptic level. However, the *in vivo* physiological stimulation approach used in this study does not allow any control on the spatial specificity of the inputs. Thus, specific experiments in which the activated synapses can be analyzed at different times after stimulation are needed to address this possibility.

### pERK ultrastructural localization and protein synthesis

It has been shown that ERK may enhance protein synthesis through phosphorylation of eIF-4E [50], and that this effect is mediated by the ERK-dependent kinase Mnk1 [51]. Interestingly, translational activation initiated by ERK signalling seems to be crucial in the control of cell-wide and somatodendritic protein synthesis underlying the stabilization of the late phases of synaptic plasticity and memory formation [52], [53]. The presence of pERK-immunolabelling on the external face of the RER (Fig 2A,B) is in agreement with a role of ERK in protein synthesis processes occurring in neurons. To our knowledge, this is the first *in vivo* demonstration that ERK activation can be targeted to the RER, thus adding further evidence for a role of the Ras/ERK pathway in the translation of mRNA. In addition, the presence of activated ERK in the core of dendritic spines supports a role of this molecule as a key regulator in local protein synthesis. It has been shown that translation of mRNA residing in dendrites and at synaptic sites plays an important role in long-lasting forms of synaptic plasticity and memory [54], [55]. Thus, persistent activation of local ERK could be explained with the need of sustained synthesis of new proteins at potentiated synapses, which are needed for the structural changes associated with the late-phase of synaptic plasticity. It will be of extreme interest to further characterize the presence at the synaptic level of other components of the translational machinery, and whether *in vivo* synaptic activity may alter their distribution and function.

### Early and late effects of ERK activation during synaptic plasticity

Many studies on the mechanisms underlying LTP have led to the idea that synaptic plasticity undergoes an initial phase that is independent from gene transcription. This early process is mediated by activity-dependent mechanisms that occur peripherally at the synaptic level and involve covalent modifications of pre-existing proteins and local protein synthesis [56]. Experiments performed in several brain structures, including the developing visual cortex and the hippocampus, have shown that ERK is rapidly activated after the plasticity inducing stimulus [2], [33], [34], and that ERK activation is crucial also for the initial phases of LTP [57]. These observations suggested that ERK involvement in LTP mechanisms is not only due to its ability to translocate to the nucleus and regulate the transcription of plasticity-related genes, but also to a local activity at the level of synaptic connections. A possible molecular model underlying the role of ERK in the different temporal phases of synaptic plasticity consists in ERK being activated immediately after synaptic stimulation. Phosphorylated ERK could then translocate to the nucleus thus disappearing from synapses [21]. A similar model has been recently suggested for importins, a class of molecules that could act as carriers of retrograde messengers that signal from the synapse to the nucleus [58]. Importins immunoreactivity has been found at distal hippocampal neuronal processes, including synaptic sites, in basal conditions. In response to neuronal activation, importins translocate into the nucleus, meanwhile their immunoreactivity decreases at dendritic and synaptic levels [59]. Our in vivo study suggests a different model for spatio-temporal dynamics of visually activated ERK. Indeed, phosphorylated ERK was present simultaneously at synaptic and soma level at early times after stimulation and its disappearance from these two compartments followed the same kinetics. Thus, although it cannot be excluded that synaptically activated ERK may translocate to the cell body upon neuronal stimulation, these data clearly indicate that pERK remains persistently elevated at synaptic sites, where it may play an important role in modulating local processes of plasticity. Taken together, our data suggest a model for the existence in a nerve cell of spatially and functionally separated sites in which ERK can be activated by the same stimuli at the same time. Thus, the involvement of ERK in early or late events occurring in the different neuronal compartments is not a consequence of different kinetics of ERK activation in the different subcellular compartments, but is likely due to differences in the molecular targets activated by ERK in distinct cell compartments.

## Supporting Information

Figure S1 We controlled the specificity of pERK immunolabelling by analysing brain sections after SL327 injection. A and B show confocal images of pERK immunofluorescence in the visual cortex of a rat exposed to light for 2.5 minutes after 3 days of dark rearing and injected with SL327 (B), and of another rat that received the same visual stimulation and vehicle injection (DMSO) as a control. It is evident that in the SL327 injected animal pERK immunoreactivity is completely abolished. We further tested the distribution of pERK using a polyclonal antibody on visual cortical sections of a normally reared rat. As shown in C, p-ERK immunoreactivity is present in the soma and in the apical (arrow) and basal dendrites (arrowhead) of cortical pyramidal neurons that are localized in layer II/III of the primary visual cortex. This pattern of immunolabelling is virtually identical to the one that we observe using a monoclonal antibody against pERK (see Fig. 1A). Scale bar: in A,B = 60 µm; in C = 20 µm.
(3.49 MB TIF)

Figure S2 To assess the reliability of pERK immunogold localization at synapses, we analyzed consecutive thin sections of the primary visual cortex. Micrographs in A′-A′ illustrate the consistency of labeling in serial sections of an axo-spinous synapse. Immunogold particles decorate a presynaptic terminal (asterisks) in all three sections whereas the juxtaposed postsynaptic spine does not show any labeling. B′-B′ show another example of the reliability of pERK immunogold labeling. An unlabelled axo-spinous synapse (arrows) adjacent to a pERK-positive dendritic profile is shown in three serial thin sections cut through layer I of the visual cortex of a rat. Scale bars: 200 nm
(2.99 MB TIF)
