# Supplementary figures and images for: Visual Stimulation Activates ERK in Synaptic and Somatic Compartments of Rat Cortical Neurons with Parallel Kinetics
Source: PLoS One. 2007 Jul 11;2(7):e604. doi: 10.1371/journal.pone.0000604 (PMC1899229; doi:10.1371/journal.pone.0000604)

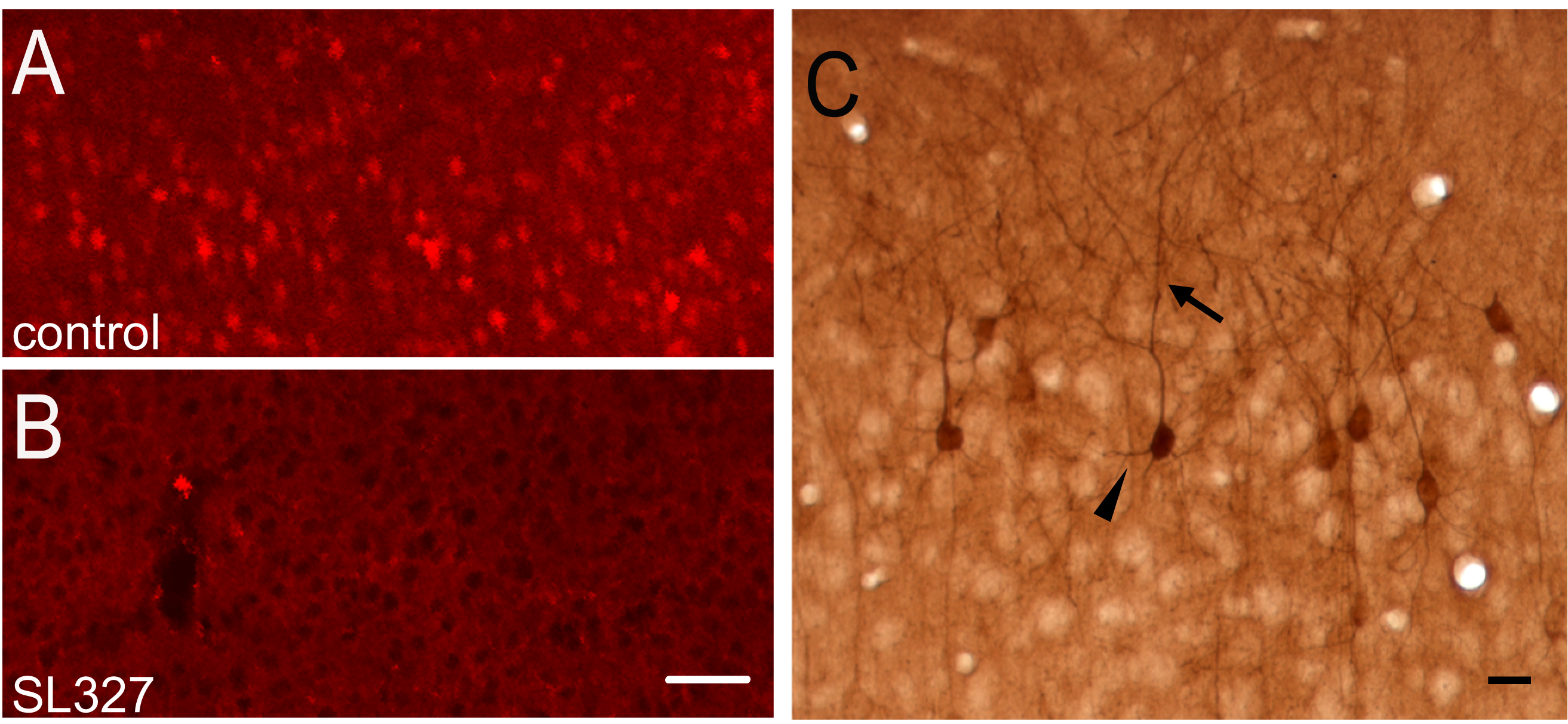

Supplement: Figure S1 — We controlled the specificity of pERK immunolabelling by analysing brain sections after SL327 injection. A and B show confocal images of pERK immunofluorescence in the visual cortex of a rat exposed to light for 2.5 minutes after 3 days of dark rearing and injected with SL327 (B), and of another rat that received the same visual stimulation and vehicle injection (DMSO) as a control. It is evident that in the SL327 injected animal pERK immunoreactivity is completely abolished. We further tested the distribution of pERK using a polyclonal antibody on visual cortical sections of a normally reared rat. As shown in C, p-ERK immunoreactivity is present in the soma and in the apical (arrow) and basal dendrites (arrowhead) of cortical pyramidal neurons that are localized in layer II/III of the primary visual cortex. This pattern of immunolabelling is virtually identical to the one that we observe using a monoclonal antibody against pERK (see Fig. 1A). Scale bar: in A,B = 60 µm; in C = 20 µm. (3.49 MB TIF) [file pone.0000604.s001.tif]

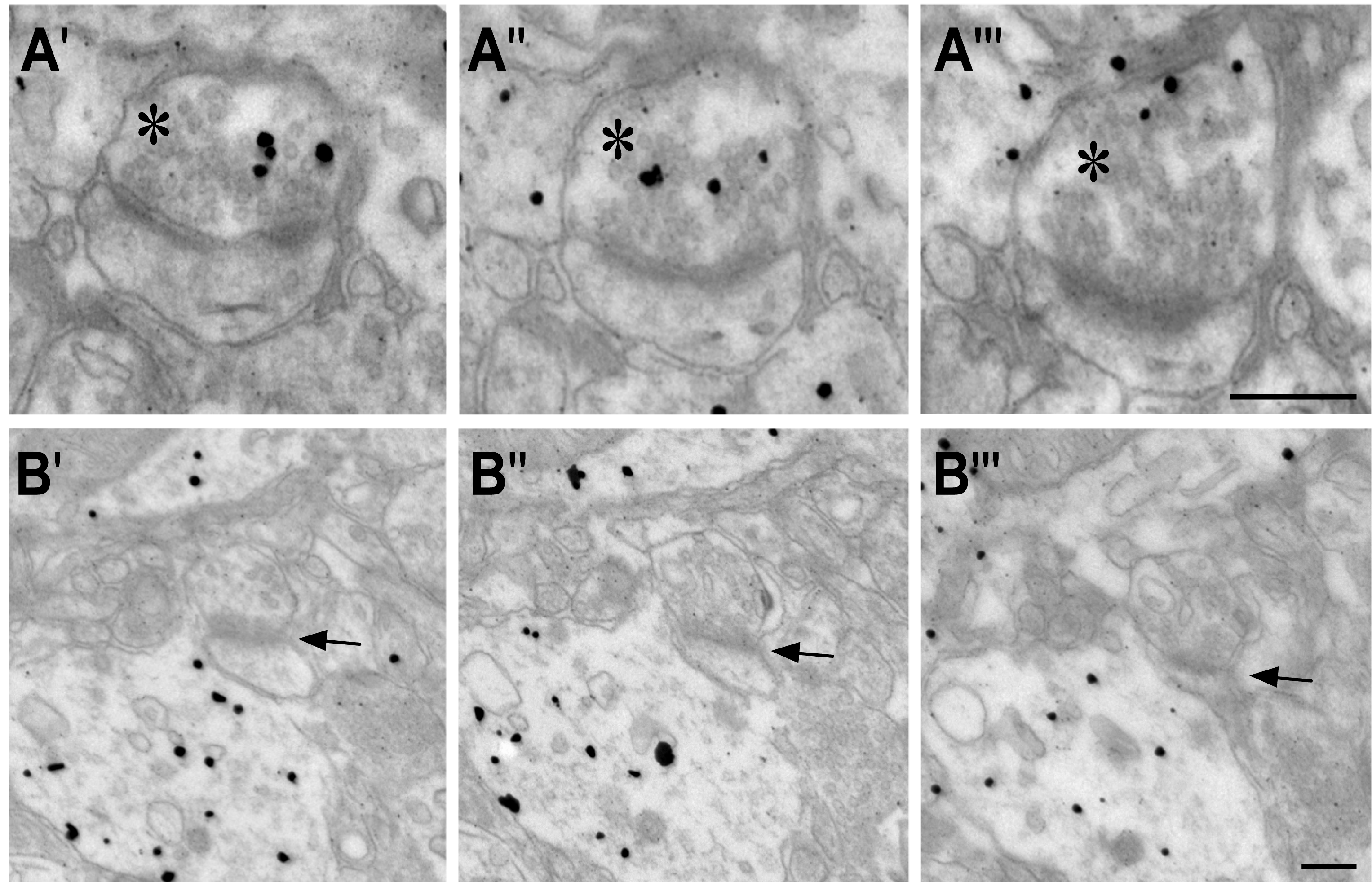

Supplement: Figure S2 — To assess the reliability of pERK immunogold localization at synapses, we analyzed consecutive thin sections of the primary visual cortex. Micrographs in A′-A′ illustrate the consistency of labeling in serial sections of an axo-spinous synapse. Immunogold particles decorate a presynaptic terminal (asterisks) in all three sections whereas the juxtaposed postsynaptic spine does not show any labeling. B′-B′ show another example of the reliability of pERK immunogold labeling. An unlabelled axo-spinous synapse (arrows) adjacent to a pERK-positive dendritic profile is shown in three serial thin sections cut through layer I of the visual cortex of a rat. Scale bars: 200 nm (2.99 MB TIF) [file pone.0000604.s002.tif]
